# Tailored treatments in inborn errors of immunity associated with atopy (IEIs-A) with skin involvement

**DOI:** 10.3389/fped.2023.1129249

**Published:** 2023-03-22

**Authors:** Carmela Giancotta, Nicole Colantoni, Lucia Pacillo, Veronica Santilli, Donato Amodio, Emma Concetta Manno, Nicola Cotugno, Gioacchino Andrea Rotulo, Beatrice Rivalta, Andrea Finocchi, Caterina Cancrini, Andrea Diociaiuti, May El Hachem, Paola Zangari

**Affiliations:** ^1^ Academic Department of Pediatrics (DPUO), Research Unit of Clinical Immunology and Vaccinology, IRCCS Bambino Gesù Children's Hospital, Rome, Italy; ^2^ Department of Systems Medicine, University of Tor Vergata, Rome, Italy; ^3^ Department of Neuroscience, Rehabilitation, Ophthalmology, Genetics, Maternal and Child Health (DINOGMI), University of Genoa, Genoa, Italy; ^4^ Dermatology Unit and Genodermatosis Unit, Genetics and Rare Diseases Research Division, IRCCS Bambino Gesù Children's Hospital, Rome, Italy

**Keywords:** skin involvement, eczema, tailored treatment, biologics, inborn errors of immunity

## Abstract

Inborn errors of immunity associated with atopy (IEIs-A) are a group of inherited monogenic disorders that occur with immune dysregulation and frequent skin involvement. Several pathways are involved in the pathogenesis of these conditions, including immune system defects, alterations of skin barrier and metabolism perturbations. Current technological improvements and the higher accessibility to genetic testing, recently allowed the identification of novel molecular pathways involved in IEIs-A, also informing on potential tailored therapeutic strategies. Compared to other systemic therapy for skin diseases, biologics have the less toxic and the best tolerated profile in the setting of immune dysregulation. Here, we review IEIs-A with skin involvement focusing on the tailored therapeutic approach according to their pathogenetic mechanism.

## 1. Introduction

Inborn errors of immunity (IEIs) include more than 400 inherited disorders causing specific perturbations of immune development and function (1). The knowledge about the IEIs is increasing over time and it has been definitely demonstrated that severe allergic inflammation may be the initial presentation of the immune system dysregulation. Inborn errors of immunity associated with atopy (IEIs-A) also defined primary atopic disorders (PADs) have been categorized for the first time in 2018 as a subgroup of IEIs characterized by allergy or atopy manifestations (2). Skin involvement is frequently present in these conditions and may present with eczema, urticaria and erythroderma. Among these skin manifestations, atopic dermatitis (AD) is the most common form of eczema, characterized by pruritus, skin inflammation and chronic/relapsing course. IEIs-A diagnosis may be challenging in the setting of skin disorders, and their management and outcome can be widely different. In the last decades, the expanding employment of next-generation sequencing (NGS) has resulted in the identification of novel candidate disease genes and enabled the molecular diagnosis of an increasing number of patients with IEIs. The identification of specific gene defects in IEIs-A has the opportunity to inform on possible therapeutic targets and personalized approaches.

In the present review, we focus on IEIs-A with skin manifestations and in particular on their pathogenetic mechanism and the therapeutic approach targeting the underpinning immune defect. Literature review was performed using Pubmed, Scopus,Web of Science databases and ClinicalTrials.gov, recovering publications on IEIs with atopic manifestations. The search approach was performed using a free-text search (keywords: inborn errors of immunity, primary immunodeficiency with atopy and allergy, atopic disorders, tailored therapies, biologic drugs). We searched recent articles published up to December 2022.

## 2. Pathogenetic mechanism and treatment of IEIs-A with skin involvement

IEIs-A include different genetic disorders with several pathogenetic pathways responsible for generating an atopic environment, possibly associated with elevation of serum total immunoglobulin (Ig) E. The major mechanisms involved in the genesis of atopy, range from immune system defect and alterations of skin barrier to metabolism perturbations (Table 1).

**Table 1:** Classification of IEIs-A.

Pathogenetic mechanism	Gene mutation	Skin involvement	Immunological phenotype	Conventional therapy	Tailored therapy
Cytoskeletal abnormalities	WAS	Eczema	↑ IgE level, eosinophilia, Thrombocytopenia	HSCT, immunosuppressive drugs (CST, RTX, CP, AZA, CNI), TT	Dupilumab, omalizumab, GT
	ARPC1B	Eczema	↑ IgE and IgA level, eosinophilia, lymphopenia, thrombocytopenia	HSCT, TT	Dupilumab
	DOCK8	Eczema	↑ IgE level, eosinophilia, ↓ IgM level,↓T cells, ↓ Th17 cells, ↑ B cells, ↓switched memory B cells	HSCT, TT	Dupilumab, omalizumab
Impaired T cell receptor signaling	CARD11 LOF	Atopic dermatitis	↑ IgE level, eosinophilia, normal or ↓ B cells, normal/↓IgG,↓T cell proliferation,↓NF-*κ*B phosphorylation/IκB*α* degradation	TT	Glutamine supplementation,dupilumab
	CARD14 LOF/GOF	Atopic dermatitis, psoriasis, pityriasis rubra pilaris	↑ IgE level, eosinophilia,	NA	NA
	MALT1	Eczema	↑ CD3 + and CD4+, ↓ T cell proliferation, ↓ NF-κB phosphorylation, ↓IκBα degradation, ↓IL-2 secretion ↑ IgE level	HSCT, TT	NA
Skin barrier dysfunction	SPINK5	Ichtyosis, atopic dermatitis	↑ IgE level, eosinophilia, ↓ memory B cells	TT, calcipotriol, AAT, OR, UVB, IVIG	TNF inhibitors, dupilumab, omalizumab, ustekinumab, GT
	DSG1	Atopic dermatitis, psoriasiform dermatitis	↑ IgE level, eosinophilia	NA	Ustekinumab, sekukinumab
	FLG	Atopic dermatitis	↑ IgE level, eosinophilia	TT	FLG replacement therapy
	CDSN	Atopic dermatitis, ichthyosiform erythroderma	↑ IgE level, eosinophilia	Kallikrein inhibitors, TT	GT
Mast cell deregulation	PLCG2	Cold urticaria, skin granulomas	↓ IgM and IgA, ↑ IgE level, ↓ memory B cells	CST, antihistamines, dapsone and hydroxychloroquine	Omalizumab, IL1 and TNF inhibitors
Metabolic disturbance	PGM3	Atopic dermatitis	↑ IgE level, normal/↑ IgG and IgA, T cell lymphopenia, ↓B cells and memory B cells, neutropenia	HSCT, Galactose/GlcNAc/uridine supplementation, TT	NA
T cell repertoire restriction	RAG1/RAG2	Erythroderma	↑ IgE level, eosinophilia, lymphopenia, T-B-NK+	HSCT, immunosuppressive drugs (CsA, CST), TT	Dupilumab, GT, GE
	ZAP70	Erythroderma	Eosinophilia, ↓CD8	HSCT, immunosuppressive drugs (CsA, CST), TT	NA
	IL7-R	Erythroderma	T-B + NK+	HSCT, immunosuppressive drugs (CsA, CST), TT	GT
	IL2RG	Erythroderma	↑ IgE level, eosinophilia, T-B + NK-	HSCT, immunosuppressive drugs (CsA, CST), TT	NA
	LIG4	Erythroderma	↑ IgE level, eosinophilia, T-B-NK+	HSCT, immunosuppressive drugs (CsA, CST), TT	NA
	DCLRE1C	Erythroderma	↑ IgE level, eosinophilia, T-B-NK+	HSCT, immunosuppressive drugs (CsA, CST), TT	NA
	Atypical Complete DiGeorge Syndrome 22q11del	Erythroderma	↓T cells,↓TREC, oligoclonal T-cell expansion	CTT, immunosuppressive drugs, TT, ThT	NA
	CHD7	Erythroderma	↓T cells,↓TREC	NA	NA
	FOXN1	Erythroderma, total alopecia	↓T cells	HSCT, ThT	NA
	TBX1E1C	Erythroderma	↓T cells,↓TREC	NA	NA
Cytokine signalling defects	STAT3 LOF	Eczema	↑ IgE level, eosinophilia, lymphopenia, ↓ TH17 level, ↓ memory B cells	HSCT, immunosuppressive drugs (CST, tacrolimus, CsA), TT	Dupilumab
	ZNF341	Eczema	↑ IgE and IgG level, ↓ TH17 level and NK cells, ↓ memory B cells	HSCT, TT	Dupilumab
	TGFBR	Eczema	↑ IgE level, eosinophilia	NA	NA
	STAT5b	Eczema	↑ IgE level, moderate lymphopenia, ↓ NK and T cells, ↑ B cells and IgG level	NA	NA
	IL6ST	Eczema	↑ IgE level, eosinophilia, ↓ Th17 cells, ↓ memory B cells	NA	NA
	TYK2	Psoriasis-like dermatitis, atopic dermatitis	↑ IgE level (incostant)	NA	JAK inhibitors
	STAT6 GOF	Atopic dermatitis	↑ IgE level, eosinophilia	NA	Dupilumab, JAK inhibitors
	ERBIN	Eczema	↑ IgE level, eosinophilia, ↑ Treg	NA	NA
	IL6RA	Eczema, skin abscess	↑ IgE level, normal/↓ IgM, G, A, ↓ switched memory B cells	NA	NA
Regulatory T Cell Defects	FOXP3	Eczema	↑ IgE and IgA levels, eosinophilia, ↓ Treg, normal CD4/CD8 T cells	HSCT, immunosuppressive drugs (CNI, CsA, sirolimus, tacrolimus, CST), mTOR inhibitors	Dupilumab, GT, GE

CST, corticostreoids; CsA, ciclosporin A; HSCT, hematopoietic stem cell transplantation; GT, gene therapy; GE, genome editing; RTX, rituximab; CP, cyclophosphamide; AZA, azathioprine; MMF, mycophenolate mofetil; AAT, alpha-1-antitrypsin; OR, oral retinoids; TT, topical treatment (moisturizers, topical corticosteroids, topical calcineurin inhibitors); IVIG, IntraVenous ImmunoGlobulin; CTT, cultured thymus tissue; ThT Thymic transplant.

### 2.1. Impaired T cell receptor signaling and cytoskeletal remodeling

#### 2.1.1. WAS

Wiskott-Aldrich syndrome (WAS) is an X-linked recessive disorder characterized by thrombocytopenia, infections, eczematous rash and a high risk of developing malignancy and autoimmune diseases. The illness is due to mutations in *WAS* gene which encodes WAS protein (WASp), involved in cell signaling and remodeling of cytoskeleton in hematopoietic cells. The WASp is crucial for T cell proliferation, differentiation and survival. WASp deficient T lymphocytes show gene transcription alterations of Th1 cytokines, leading to a skewed Th2 response (3). The restriction of T cell receptor-repertoire diversity has been shown to contribute to this immune dysregulation (4). WAS patients present normal frequency of regulatory T (Treg) cells, but their function is impaired as demonstrated by low interleukin (IL)-10 production potentially predisposing to pathological inflammation and autoimmunity (5). It has been reported that WASp is also involved in the development of regulatory B (Breg) cell, affecting the equilibrium and migration of Treg and Th17 cells during the inflammatory state (6). High levels of Th2 and Th17 cytokines have been found in the skin, as well as the itch-associated molecules (7) both contributing to an inflammatory environment. Moreover, high eosinophils and serum IgE levels are often present (3).

Eczema is found in about 80% of WAS patients, with the characteristics of an early onset, severe and widespread AD, accompanied by petechiae and purpura due to the associated thrombocytopenia.

WAS treatment strategies consist of supportive measures, hematopoietic stem cell transplantation (HSCT) and gene therapy. Immunosuppressive/immunomodulatory drugs to control autoimmune diseases linked to WAS include corticosteroids, intravenous immunoglobulin (IVIG), rituximab, cyclophosphamide, azathioprine, and calcineurin inhibitors (8). With regard to the dermatitis the treatment is based on topical emollients, corticosteroids and according to some authors antiseptic baths (3).

Immunosuppressive drugs (corticosteroids, cyclosporine) are usually administered to control immune dysregulation signs. Anakinra, a human IL-1 receptor antagonist, has been administered with good results, suggesting an involvement of the innate immunity in the generation of auto inflammatory manifestations in WAS patients (9). It has been also described a partial response to omalizumab, a humanized recombinant monoclonal anti IgE antibody, in a genetically confirmed child with WAS and atypical clinical manifestations. The patient presented a history of diffuse pruritic eczema resistant to conventional systemic immunosuppressive therapy, which improved after three subcutaneous injections of omalizumab with concomitant topical steroid (10).

Patients with classic WAS are prone to autoimmune disorders and lymphoma or other malignancies (11). However, the clinical phenotype of WAS is variable and there are patients with less severe symptoms who survive childhood and therefore do not require transplantation, especially in cases due to hypomorphic variants in the *WAS* gene (12). In the classic form of WAS, the gold-standard treatment is represented by bone marrow transplantation (13–15). The outcomes of children undergoing HSCT are optimal, with a survival rate of more than 97%. In contrast, the few patients who did not underwent HSCT did not reach adulthood (16). Age at HSCT seems to be the only factor significantly associated with overall survival (OS), in fact, patients below 5 years of age have higher OS compared with those who were older then 5 years at the time of HSCT (5-year OS: 94% vs. 66%, respectively) (14). Conversely, OS is not significantly associated with conditioning regimen intensity, donor type, hematopoietic cell source, disease severity, and WASp expression. Full chimerism seems to decrease the incidence of post-HSCT autoimmune diseases and chronic inflammation. Of note, some clinical features of the syndrome, such as AD, may persist in a percentage of patients after transplantation (17).

It was recently described a WAS patient who developed graft-versus-host disease (GvHD) following HSCT. Skin lesions and a high titer of IgE persisted after the use of immunosuppressive treatment. Therefore, a Th2 pathogenesis has been hypothesized, and dupilumab, a monoclonal antibody that inhibits IL-4 and IL-13 signaling, was started with significant clinical benefit (18).

Gene therapy is another effective and safe treatment for WAS providing an adequate immunological reconstitution and control of autoimmunity in most patients (13). Currently, the use of lentiviral vector gene therapy showed great efficacy in patients with WAS who do not have a compatible donor (19).

#### 2.1.2. ARPC1B

Atopic manifestations are described in the platelet abnormalities with eosinophilia and immunomediated inflammatory disease (PLTEID) due to biallelic variants of the actin-related protein 2/3 complex subunit 1B (*ARPC1B*) gene. PLTEID patients present a broad spectrum phenotype resembling WAS phenotype including severe inflammatory state, lymphoproliferation, purpura, bleeding and immunodeficiency characterized by eczema, severe infections and early-onset vasculitis (20).

ARPC1B protein is a component of the actin-related protein 2/3 complex (Arp2/3) and together with WASp regulate cytoskeletal remodeling of actin and the DNA damage response (21).

Auto inflammatory manifestations of ARPC1B patients are potentially controlled by immunosuppressive therapy such as corticosteroids, mofetil mycophenolate, and rapamycin. Conversely, the use of anti-TNF drugs led to unsatisfactory results. Given the early onset symptoms and the severity of comorbidities, HSCT is currently the only curative treatment (22). In seven *ARPCB1* patients, allo-HSCT has been associated with a high survival rate with limited post-transplant morbidity (23). At a median follow-up of 19 months, 6 out of 7 patients are alive and disease-free.

In selected cases, specifically in presence of atopic disorders, biological drugs targeting Th2 pathway could be used. We recently reported a substantial improvement of eczema after starting dupilumab in an ARPC1B child whose phenotype was characterized by frequent infections, thrombocytopenia, elevated eosinophils, IgA and IgE levels, vasculitis, colitis and severe dermatitis refractory to conventional medical therapy. At the age of 10 years, she received dupilumab with significant improvement of dermatitis and itchiness (21).

#### 2.1.3. DOCK8

Dedicator of cytokinesis 8 (*DOCK8*) encodes a protein highly expressed in lymphocytes behaving as actin cytoskeleton regulator. Biallelic loss-of-function (LOF) *DOCK8* mutations result in a combined immunodeficiency characterized by atopy, severe infections, autoimmunity, and malignancy. *DOCK8* deficiency impairs the survival, function and migration of immune cells and it impacts both innate and adaptive immune responses. Adaptive immune response is affected through several mechanisms. Among them the main mechanism is related to the impaired actin cytoskeleton rearrangement that causes a defective immune synapse formation. This contributes to impaired B, T and NKT cell survival and long-lived memory responses. Moreover, NK and CD8 cells show an impaired effector activity (24). Naïve *DOCK8*-deficient CD4+ T cells display increased differentiation towards the Th2 cells and a higher proportion of activated cells producing Th2 cytokines when compared to controls (24).

Few cases of pediatric patients with *DOCK8* mutation treated with dupilumab are described so far. Two female patients, 10 and 11.5 years old respectively, obtained a substantial clinical benefit from dupilumab administration after only one month of treatment. The itchiness was much improved and also secondary skin infections were reduced, without increase in systemic infections. Serum IgE levels decreased significantly after treatment (25).

The use of omalizumab in an adult patient with *DOCK8* mutation has been described with an improvement of skin lesions (26).

Biological drugs are a viable alternative to improve the skin manifestations, and consequently the quality of life, in patients awaiting HSCT, that remains the only resolutive treatment.

An international survey of 136 *DOCK8* transplanted patients patients showed an OS of 87% at 10 years, 47% at 20 years, and 33% at 30 years (27). A multicenter retrospective study of 22 patients reported an OS of 89% after matched related HSCT and 81% after unrelated HSCT (28).

#### 2.1.4. MALT1

Mucosa-associated lymphoid tissue lymphoma translocation protein 1 (MALT1) is a paracaspase assembled with B cell CLL/lymphoma 10 (BCL10). Following receptor stimulation, BCL10-MALT1 binds to a caspase recruitment domain (CARD) family proteins such as CARD9, CARD10 or CARD11, forming the CARD-BCL10-MALT1 (CBM) complex. It binds antigen receptors activating the signaling of the NF-*κ*B, JNK, and mTORC1 pathways. The CBM complex and consequently *MALT1,* have a crucial role in activation, survival, proliferation and metabolism of lymphocytes. Germline LOF variants in *MALT1* clinically present recurrent infections, oral and intestinal mucosal involvement, dermatitis and failure to thrive. The impaired CARD-dependent signaling observed in keratinocytes of *MALT1* deficient patients could alter the skin barrier and lead to an increase risk of skin infections as well as dermatitis (29). Since the relevance of the CBM complex in the development of several diseases, targeted drugs acting on this pathway are recently attracting research interest. In particular, MALT1 inhibitors are considered specific and efficient drugs that might be finally promising options for the therapy of malignancies and diseases associated with lymphoproliferation (30).

Of note, *MALT1* deficiency has been successfully treated with HSCT (31–34).

#### 2.1.5. CARD11

CARD11 is a multidomain scaffold protein needed to induce NF-kB, JNK, and mTOR following antigen receptor stimulation. Germline *CARD11* mutations are mainly associated to three different IEIs: *CARD11* deficiency, B cell expansion with NF-kB and T cell anergy (BENTA) and CARD11-associated Atopy with Dominant Interference of NF-kB Signalling (CADINS). CADINS is due to heterozygous LOF dominant negative variants of the gene. Many mutations associated with CADINS also downregulate TCR-mediated mTORC1 activation, probably due to a reduction in the glutamine uptake. TCR signaling abnormalities cause an impaired T cell proliferation/activation, an increase of Th2 cytokines and a decreased of Th1 cytokines production. Clinically, patients with CADINS usually present early onset atopy (AD, asthma, food allergies, and eosinophilic esophagitis), recurrent viral skin and respiratory tract infections (35). In a recent single center cohort study, AD and skin infections ameliorated or even resolved during adolescence, suggesting a spontaneous dermatological improvement over time (36).

Glutamine is involved in immunomodulatory functions, but its impact on regulating T-cell function is still unclear (37). In infants with low birth weight and atopy, glutamine supplementation has been studied with a promising reduction in AD (38). *CARD11* mutations prevent the upregulation of the glutamine transporter ASCT2 and mTORC1 activating cell proliferation, thus glutamine supplementation has been proposed to improve atopic manifestations in *CARD11* or related genes mutations. Interestingly, amino acid supplementation could modulate the immune metabolism and also improve AD (39).The Th2/Th1 imbalance in *CARD11* deficient patients indicates that dupilumab might be useful in controlling AD. Case reports of CADINS patients with severe AD successfully treated with dupilumab, without side effects, have been described (40–42).

#### 2.1.6. CARD14

*CARD14* induces the NF-*κ*B and mitogen-activated protein kinase (MAPK or MAP kinase) signaling through BCL10 and MALT1, upregulating pro-inflammatory genes. While upregulation of *CARD14* gives a skin picture overlapping with psoriasis (43) and atypical juvenile pityriasis rubra pilaris (44, 45), its downregulation is associated with AD, increased risk of skin infection and also dysregulating cutaneous inflammation. Dominant LOF mutations in *CARD14* are associated with severe AD, impaired NF-kB cascade, and dysregulation of innate immunity mediators involved in AD pathogenesis (46). The upregulation of *CARD14* lead to excessive expression of NF-kB-responsive genes and initiate the recruitment of the inflammatory cells, including dendritic cells and T cells producing IL-23 and IL-17/IL-22 respectively (47). In line with this, ustekinumab, an inhibitor that targets both IL-12 and IL-23 cytokines, proved to be a successful treatment in an increasing numbers of patients with *CARD14* GOF mutations (45, 48–51). Since the role of *CARD14* in both AD and psoriatic diseases, targeted therapies in these patients need to be considered with caution. To our knowledge, there are no clinical reports on the application of targeted therapy in patients with *CARD14* LOF mutations.

### 2.2. Skin barrier dysfunction

#### 2.2.1. SPINK5

The Comèl-Netherton syndrome (NS) is an inherited disease due to biallelic mutations in the serine protease inhibitor Kazal-type 5 (*SPINK5*) gene, encoding for inhibitor lympho-epithelial Kazal-type-related inhibitor (LEKTI) that regulates many proteolytic events including the cleavage of desmosomal connections (52). *SPINK5* is necessary to maintain skin barrier integrity. In fact, LEKTI deficiency results in defective barrier function (1). NS patients may present with erythroderma or ichthyosis linearis circumflexa as well as typical hair anomalies called trichorhexis nodosa (bamboo hair) (53). Skin and hair defects persist over time, but the disorder usually ameliorates with age (52). The mortality rate is high in the first years of life due to potentially fatal complications (54). In NS epidermidis, the exaggerated protease activity causes the overexpression of proinflammatory, and proallergic cytokines. These molecules drive Th2 cytokine production and lead to atopy and elevated IgE level (55, 56). The IL-17/IL-23 pathway was found to be a predominant signaling axis in NS (57). Recently, 2 endotypes of NS are distinguished on the basis of multiomics analysis: NS with typical ichthyosis linearis circumflexa (NS-ILC) and scaly erythroderma (NS-SE). In NS-ILC, a Th2- complement driven immune response was observed with neutrophil infiltration and complement activation. In NS-SE, a type I IFN*γ*-driven inflammatory axis appeared prevalent (58).

Conventional treatments include skin care along with supportive care. These interventions can improve cutaneous symptoms without restoring the skin integrity entirely. The use of low-potency corticosteroids, as well as topical calcineurin inhibitors, have shown beneficial effects in some patients (55) but, due to the barrier defect, significant cutaneous absorption cannot be ensured. Over the years, several groups described a significant improvement of the cutaneous signs and symptoms in NS children treated with monthly IVIG (59–64). It was hypothesized that IVIG treatment in NS patients decrease the inflammation by downregulating type 17 inflammation and restoring immune homeostasis (65). Therefore, a trial of IVIG may be considered in severe NS patients.

The administration of kallikrein inhibitors consists of a protein replacement therapy and would be a more etiological treatment. It appears to ameliorate symptoms of NS in animal models (66) and promising results have been observed also in humans (67).

Gene therapy using a lentiviral vector encoding the *SPINK5* gene is under investigation (68, 69). A recent trial proposed grafting autologous epidermal sheets derived from genetically modified skin stem cells to release LEKTI protein in NS patients. Results are still not available (70).

Several case reports showed a clinical improvement in adults and children treated with dupilumab (66, 71–73). Currently, a randomized clinical trial evaluating the efficacy and safety of dupilumab in NS patients is under recruitment (74).

Omalizumab administration reduced skin and mucosal symptoms in a 20-year-old patient with NS (75).

A significant skin improvement was shown in a young adult with NS who received ustekinumab (57). Furthermore, promising results came from the use of monoclonal antibodies against IL- 17 called ixekizumab and secukinumab, TNF-α inhibitors (eg, infliximab) and anakinra to control the inflammatory skin lesions in NS (65, 76–78). The different therapeutic responses observed after inhibition of the IL-17 axis suggest that different pathways may contribute to NS pathogenesis (79). Finally, the recent discovery of two NS endotypes could inform new therapeutic approaches (58).

## 3. Other genes

Filaggrin, a filament-aggregating protein (FLG) is a key protein of the stratum corneum. LOF *FLG* mutations or mutations causing a decrease in *FLG* copy number are strongly associated with AD confirming its fundamental function for epidermal barrier integrity (80). Recently, LOF variants in the FLG gene has been recognized as a risk factor for the onset of severe manifestations of food allergy (81). FLG replacement treatment studies in murine models evidenced beneficial effects (82–84).

Desmoglein 1 (DSG1) protein belongs to the family of cadherins. Biallelic LOF mutations in *DSG1* gene cause severe dermatitis, multiple allergies and metabolic wasting (SAM) and can manifest as ichthyosiform erythroderma at birth (85). Based on an IL-17–skewed inflammatory signature revealed in these patients, the use of anti IL-17 A antibody and an IL-12/IL-23 antagonist have been proposed with promising results (86, 87).

Corneodesmosin (CDSN) is necessary for cell adhesion and skin integrity. Its expression is reduced in AD patients (88). Peeling skin syndrome (PSS) type B is a rare autosomal recessive disease caused by mutations in the *CDSN* gene. It is characterized by congenital ichthyosiform erythroderma and skin exfoliation along with elevated serum IgE. The use of antihistamines and kallikrein inhibitors have been proposed based upon the observation *in vitro* that histamine attenuates the expression of desmosomal proteins in human keratinocytes, and kallikreins are upregulated in type B PSS (89). Recently, *in vitro* studies for a protein replacement therapy in PSS patients showed encouraging results (90).

### 3.1. Mast cell deregulation

Germline mutations in two different genes called *PLCG2* and *ADGRE2* which encode for Phospholipase C gamma 2 and Adhesion protein-coupled receptor E2 respectively, are associated with a type of urticaria triggered by cold and vibration. Mutations in the *PLCG2* gene are associated to PLCG2-associated antibody deficiency and immune dysregulation (PLAID) and to auto inflammation and PLCG2-associated antibody deficiency with immune dysregulation (APLAID) syndrome. PLAID syndrome is characterized by early-onset cold urticaria, antibody deficiency, recurrent infections, autoimmune disease and symptomatic allergic disease (91–93). Patients with PLAID, when possible, should avoid cold triggers. Systemic corticosteroids seem to improve symptoms and partially control the disease. In addition, the use of other drugs such as antihistamines, omalizumab, dapsone, and hydroxychloroquine shows improvement in skin symptoms (94). As a future option, the use of specific inhibitors to normalize *PLCG2* function at body temperature and to avoid uncontrolled activation at cold exposure has been proposed, but no data are available (92).

### 3.2. Metabolic disturbance

Hypomorphic phosphoglucomutase 3 (*PGM3)* mutations with autosomal recessive transmission cause abnormal protein glycosylation and differences in the cellular metabolism. The clinical presentation is characterized by high serum IgE, atopy, neurological impairment, immunodeficiency and autoimmunity (95). In fact, it was demonstrated that altered glycosylation due to *PGM* deficiency may also affect a subset of lymphocytes (96, 97).

Substrate supplementation therapies for the defective glycosylation pathway have been proposed for several congenital disorders (98). As future therapy perspectives, a trial administering N-acetylglucosamine and uridine oral supplementation to patients with *PGM3* deficiency is still on going (99).

HSCT is known to be a curative treatment for most immunodeficiencies, but data in these conditions are limited. Two out of three children described by Pedersen et al. were successfully transplanted, while the other patient died due to infectious complications before transplantation (97).

### 3.3. T cell repertoire restriction

Omenn syndrome (Os) is an atypical presentation of severe combined immunodeficiency (SCID) with early-onset severe erythroderma and eczema, alopecia, lymphadenopathy, hepatosplenomegaly, chronic persistent diarrhea, recurrent infection and growth failure.

Many genetic defects responsible for TCR over activation can cause uncontrolled lymphocyte expansion and subsequently lymphocyte peripheral infiltration in various tissues including skin, causing tissue damage. Expansion of T-cell clones in Os is associated to Th2 differentiation, Th2 cytokines production, high levels of IgE and eosinophilia. Histological findings in erythroderma of Os are analogous to those described in severe AD. The mechanisms underlying the immunological alterations responsible for the atopic features observed in Os are still a matter of debate. Lymphopenia-induced homeostatic proliferation, poor thymic control of autoreactive lymphocytes, defective Treg and Th2 skewed response have been reported (100).

Since Os is fatal in early life, HSCT represents the first line therapy (101).

In these patients, immunosuppressive treatments such as cyclosporine and steroids are administered as bridging therapy pending HSCT. Cyclosporin, compared to steroids, can modulate T-cell functions at low concentrations, with a consequent control of immune reactivity and skin improvement (102). Finally, immunosuppression provides control of self-reactive T cells but it is often associated to side effects (103, 104). Targeted therapies downregulating Th2 response are considered as new and safe candidates for Os management. Recently, an *in vitro* model with Os CD4+ T cells showed only a mild reduction of IL-4 production after dupilumab treatment vs. control (105). This could suggest that Th2 polarized response in Os patients might not be regulated by IL-4 signaling only.

Autologous stem-cell-based gene therapy represents the new therapeutic option to treat Os patients without suitable donors. Murine models with RAG mutations treated with lentivirus-mediated gene therapy showed both immunological and clinical improvement, with a dramatic increase in naïve T cells and reduction in effector/memory T cells, and a decrease in cellular infiltration in the skin (106, 107). Currently, preclinical studies are on going to implement transgene expression and obtain stable immune reconstitution (108, 109).

### 3.4. Cytokine signaling defects

#### 3.4.1. STAT3

The prototypic hyper-immunoglobulin E syndrome (HIES) is caused by LOF autosomal dominant mutations in the signal transducer and activator of transcription 3 (*STAT3)*.

*STAT3* activity is essential in several immunological functions including differentiation of Th17 lymphocytes. STAT3 is a transcription factor modulating expression of various genes including cytokines involved in multiple pathways such as IL-6, IL-21, IL-10, IL-11, IL-22, and IL-23. This aberrant immunological transduction explains the various manifestations involving multiple organs and systems, including eczema, lung disease, skeletal and connective tissue abnormalities and vasculopathy. Indeed, the infectious phenotype in patients with *STAT3* deficiency is characterized by recurrent staphylococcal skin infections, recurrent bacterial pneumonia and chronic mucocutaneous candidiasis. Interestingly, despite high IgE levels, patients have low rates of allergy and anaphylaxis due to lower affinity of IgE for allergens (110). The skin involvement differs from common AD for early onset and other characteristic signs, such as hyperkeratosis of facial skin, retro auricular fissures, and severe folliculitis (111).

*STAT3*-deficient patients benefit antibiotic prophylaxis to prevent both dermatological and pulmonary infection. Antifungal prophylaxis should be considered in patients with structural airway abnormalities (110). IVIG replacement showed a decrease in frequency of bacterial pneumonia and can be considered to prevent recurrent lung infection (112).

Published data on HSCT in STAT3 patients are limited and controversial. In the past years results were not encouraging, with reports of transplant failure and death (113–115).

However, recent case series and follow-up studies demonstrated clinical improvement in terms of skin and pulmonary symptoms and immunological reconstitution after HSCT (116, 117).

Conventional therapy for skin manifestations includes topical and systemic immunosuppressive drugs such as steroid, tacrolimus, and cyclosporine. Given the increased IL-4 expression observed in patients with dominant negative *STAT3* mutations, it was supposed that dupilumab might treat some clinical manifestations (118). Many reports confirmed the success of the treatment with substantial improvement of the cutaneous lesions, pruritus and IgE levels (118–121).

Omalizumab demonstrated its efficacy in many immune-mediated and autoimmune skin disorders, although its role in HIES is still being defined. Several case reports described its use in STAT3 deficient patients with successfully improvement of skin symptoms and a decrease of serum IgE during treatment (26, 122, 123). Some clinical experience also reported an improvement of pulmonary manifestations (124, 125). Omalizumab was also used in combination with co-trimoxazole and inhaled tobramycin with no recurrent pulmonary or skin infection and a considerable improvement in skin lesions (126).

#### 3.4.2. ZNF341

Patients with Zinc Finger Protein 431 (*ZNF341)* deficiency phenotypically overlap with *STAT3* deficiency. However, patients with *ZNF341* deficiency are characterized by less severe non-hematopoietic phenotypes and more severe inflammatory manifestations compared to *STAT3* deficiency (118). Patients with increased radiosensitivity and subsequent increased risk of malignancy are reported (127). Furthermore, *ZNF341* deficiency seems to influence several immune cells including monocytes and NK lymphocytes, which could contribute in the generation of atopic eczema.

It was reported significantly clinical improvement and reduced IgE level in a *ZNF341* deficient adult patient with severe AD following dupilumab administration (128)..

#### 3.4.3. TYK2

Tyrosine kinase 2 (TYK2) enzyme is a member of JAK family and is implicated in the signal transduction of many cytokines including IFN-α, IL-10, IL-6 and IL-12. *TYK2* deficiency was discovered in patients with autosomal recessive (AR) HIES. Interestingly, unlike the first patient reported, the HIES phenotype was not found by the other seven patients with *TYK2* deficiency described so far (129). However, it seems that various mutation types may influence the expression of TYK2 and promote Th2 cell differentiation, resulting in increased production of Th2 cytokines (130).

## 4. Novel genes associated with cytokine signaling defects

Since the advent of NGS, a growing number of mutations associated with cytokine signaling defects are being identified but data on targeted therapy are not yet available (131–133).

*STAT5* is essential for mast cell cytokines production, proliferation and survival. The role of STAT5B in the IgE-mediated mast cell function has been confirmed in murine models (134).

*STAT6* mediates the pathway of IL-4 and a hyperactive STAT6 signaling may alters many cellular processes including increased Th2 differentiation, Th2 cytokines production, elevated IgE levels, increased expression of receptor CD23 on B cells, recruitment of eosinophils and mast cells. This immune dysregulation causes allergic inflammation, asthma and AD (135). Two recent papers identified heterozygous GOF variants in *STAT6* characterized by early onset al.lergic phenotype, refractory AD, hyper eosinophilia, high levels of IgE and vascular anomalies of the brain (136, 137). Since the involvement of the IL-4 axis was demonstrated, the use of dupilumab could be a valid therapeutic option. One of the patients reported is currently treated with dupilumab with good clinical outcome. Moreover, the authors demonstrated that *in vitro* JAK inhibition through ruxolitinib and tofacitinib effectively contained the increased STAT6 phosphorylation in cells expressing the *STAT6* variants, proposing JAK inhibitors as a valid therapeutic approach in patients with GOF *STAT6* variants (136).

Variants in genes encoding the transforming growth factor β (TGFβ) receptor, cause Loeys–Dietz syndrome (LDS), a rare connective tissue disorder that affects the heart, blood vessels, eyes, and skeletal system. Recently, an allergic phenotype characterized by asthma, food allergy, allergic rhinitis and atopic eczema, has been described (138). LDS mutations appear to lead lymphocytes to acquire and/or maintain Th2 effector functions. It was demonstrated that patients with mutations in TGFβ showed raised levels of IgE and mild reduced IL-17 cytokine production (139).

ERBB2-interacting protein (ERBIN) is necessary for TGF-β pathway activation and its expression is related to STAT3 signaling. In fact, reduced ERBIN expression was described in patients with *STAT3* mutations. A LOF *ERBIN* mutation was recently reported causing Treg and Th2 polarization and a pathological phenotype overlapping with LDS and STAT3-HIES (140).

Many variants of the Interleukin 6 Signal Transducer (*IL6ST*) gene associated with a severe AR HIES have been identified (141). Indeed, *IL6ST* gene encodes for a co-receptor of IL-6 called GP130, which transduces the STAT3 pathway (142). Despite clinical phenotype similarities, unlike STAT3 deficiency, AR *IL-6R* deficiency does not show skeletal involvement (143).

7. Regulatory T Cell DefectsIPEX syndrome is an X-linked autoimmune disease caused by mutations in forkhead box P3 (*FOXP3*) gene. The clinical phenotype mainly includes immune dysregulation, polyendocrinopathy and enteropathy (144). FOXP3 protein is implicated in the regulation and function of Treg cells, which mediates the suppression of autoreactive T cells (145). Impaired FOXP3 expression leads to a Th2-skewed predominance. Skin involvement in IPEX syndrome is heterogeneous and can include eczematous, psoriasiform, and ichthyosiform lesions, intermittent urticaria, alopecia universalis, onychodystrophy and pemphigoid lesions.

Currently, allogeneic HSCT is the best treatment option and should be performed before organ damage develops. Long-term follow-up reports a 10-year survival of 72,8% after HSCT (146). Immunosuppressive therapy is usually administrated after transplantation. Cyclosporine A, sirolimus and tacrolimus, or steroids are the most used agents. Rapamycin demonstrated to restore Treg cell function in IPEX syndrome, improving their suppression ability (147). It was demonstrated that immunosuppressive therapies alone do not impact the disease progression, and are associated with reduced life expectancy (148).

Recently, for the first time an IPEX patient with diffuse eczema was successfully treated with dupilumab. In this case, patient's dermatitis and itching persisted without improvement despite the HSCT and immune suppressive drugs (149).

Human T cells generated by viral transduction of a transcription unit encoding FOXP3, expressed a regulatory T phenotype *in vitro* (150) and could represent a novel therapeutic approach to modulate immune responses in the setting of allergy, autoimmunity, and immunodeficiencies. Initial trials of Treg-based cell therapy for IPEX syndrome are already tested *in vitro* and in animal models with promising results, but limitations are mainly related to the lifespan of the CD4 + T cells expressing wild-type FOXP3 (151).

## 5. Discussion

In the last decade, the rapid evolution of knowledge in the diagnosis and treatment of IEIs and the recognition of atopic disorders as a frequent feature have improved our knowledge of IEIs-A. Chronic skin disease is one of the main clinical manifestations in IEIs-As, and may manifest with eczema, erythroderma and urticaria. In particular, eczema is the most frequent manifestation and it is reported in 13%–22% of IEIs patients (152, 153).

In most cases, the skin involvement is similar to that found in non-immunocompromised patients, but when observed in IEIs-As, it tends to have an earlier onset, great severity, and possible complications such as infection. Another features of eczema in patients with IEIs-A is their unresponsiveness to conventional treatment that traditionally includes moisturizers, topical corticosteroids, and calcineurin inhibitors (152).

**Figure 1:**
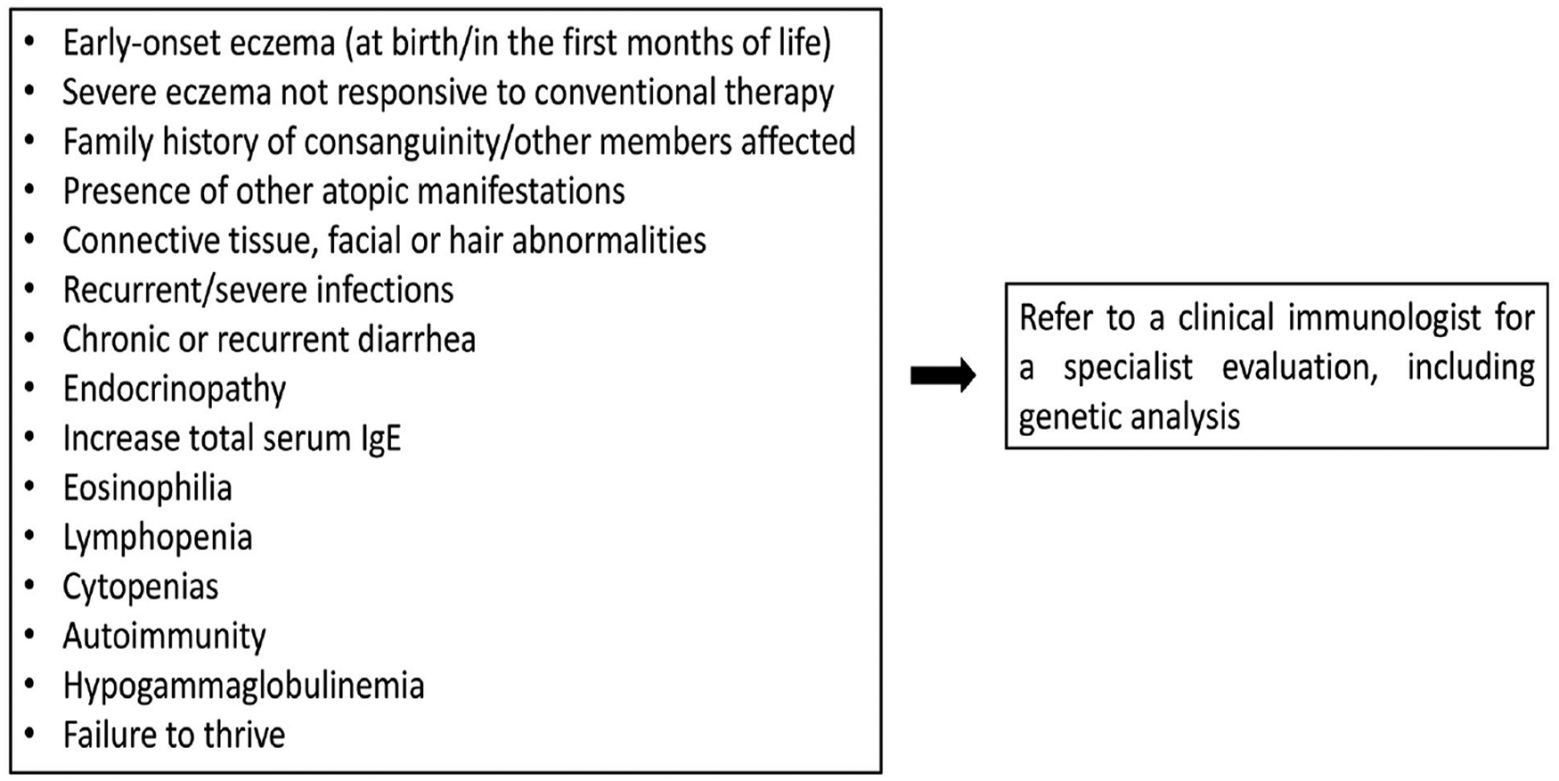
Warning signs for IEIs-A.

Figure 1 summarizes the warning signs that may guide to the diagnosis of IEIs-A.

**Figure 2:**
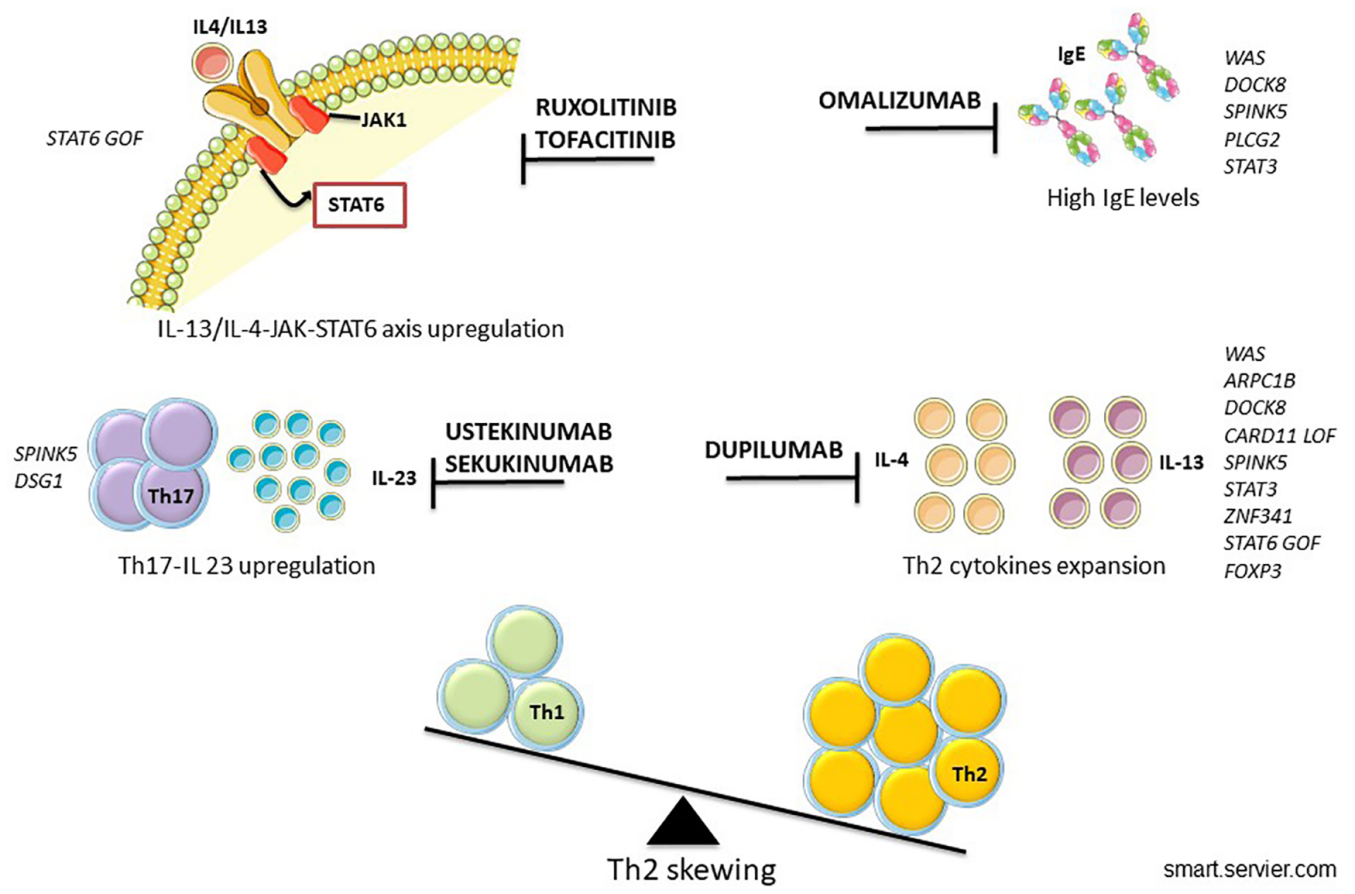
Proposed biologics in IEIs-A with skin involvement.

**Figure 3:**
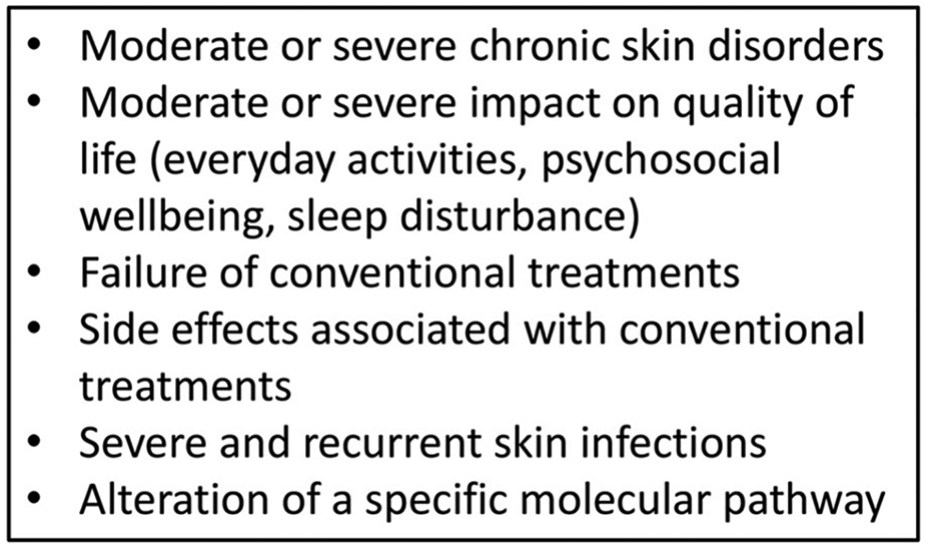
When to candidate for biologic treatment?

Continual improvements and accessibility of genetic analysis have helped to identify new IEIs-A diseases and to detect the intracellular pathway involved, allowing the possibility of precision therapy (154) (Figure 2). The goal of the tailored therapy is to use therapeutic agents to modulate dysfunctional pathways (155). Clinical evalutations to consider before starting biologic drugs are summarized in Figure 3.

Recently, biologics became an interesting and promising option to treat refractory AD. Since 2020 dupilumab represents the first option among systemic therapies, and many randomized clinical trials have proven its safety and efficacy in both children and adolescents with AD (155–158).

Dupilumab is the most studied biological drug so far. It binds IL-4R*α* inhibiting the IL-13/IL-4/STAT6 axis that includes cytokines with a crucial role in the pathogenesis of AD. In the context of IEIs-A, dupilumab has been used in many models with a Th2-skewed immune response with successful results (128). An increasing number of severe eczema treated with dupilumab IEIs-A patients are being reported in the literature (120, 159–161) and a recovery of Th1 polarization after its use is described in many cases (120).

The major advantage in treating immunocompromised patients with dupilumab is its safety profile as it does not cause further immunosuppression (162). In addition, it may be used as a bridge treatment in patients waiting for HSCT, in order to control skin infectious and inflammatory complications.

Omalizumab is currently approved for allergic asthma and chronic spontaneous urticaria. Its use in severe pediatric AD has been tested in a randomized clinical trial concluding that omalizumab is a treatment option for difficult-to-manage severe eczema in children with atopy (163). Omalizumab application in IEIs-A is limited to case reports or single clinical experience in particular in HIES with concomitant respiratory manifestations, but this application is still debated (63, 163–166).

In different forms of congenital ichthyosis the use of anti IL-17 A antibody and an IL-12/IL-23 antagonist have been proposed with promising results, based on an IL-17–skewed inflammatory signature revealed in these patients (86, 87).

In last years, many drugs involving different pathways are being studied to manage moderate and severe AD. All of the conventional treatments (steroid, cyclosporine, tacrolimus and dupilumab) inhibit the IL-13/IL-4-JAK-STAT6/STAT3 axis and the subsequent production of IL-13/IL-4 cytokines. Therefore, targeting this pathway would be a promising strategy also to develop new biologics for AD (167).

The rationale for the use of JAK inhibitors in AD is its role in controlling the transduction of the JAK-STAT signaling for Th2 cytokines. In the context of IEIs-A its use should be evaluated in selected cases where the axis is upregulated (167).

Clinical trials of new biologics for AD, already used in other diseases, include those targeting IL-13, IL-31RA, IL-33, OX40 and IL-22 (168). Their mechanism and phase of study are summarized in Table 2.

**Table 2:** Biological therapies for AD.

Tailored therapy	Inhibition target	ClinicalTrials.gov	Age	Phases	Result	Approvation
Dupilumab	IL-4Rα	Liberty AD PRESCHOOL	≥6 m and <6 y	2	skin improvement	FDA for adults with moderate-to-severe AD (2017) (169)
		NCT02612454	≥6 m and <18 y	3	NA	EU for adolescents with moderate-to-severe AD (2019) (170)
		RAD 2021	6–11 y	3	NA	FDA for children 6 to 11 y with moderate-to-severe AD (2020) (171)
Omalizumab	IgE receptor	AMB-WEI-1052-I	18–70 y	2	NA	
		OXAD	4–25 y	1	skin improvement	
		ADAPT	4–19 y	4	skin improvement	
		NCT00822783	12–60 y	4	NA	
Mepolizumab	IL-5	NCT03055195	≥18 y	2	not skin improvement	
Tralokinumab	IL-13Ra1, Ra2	ECZYTA 1	≥18 y	2	skin improvement	
		ECZTRA 2	≥18 y	3	skin improvement	
Lebrikizumab	IL-13	ADvocate1, ADvocate2	≥12 y	2	skin improvement	
Nemolizumab	IL-31Ra	NCT03989349	≥12 y	3	itch improvement	Japan for adolescents ≥ 13 y with itch associated with AD (2022) (172)
		NCT03985943	≥12 y	3	itch improvement	
		NCT03100344	≥18 y	2	itch improvement	
		NCT05056779	≥18 y	3	skin and itch improvement	
		NCT03989206	≥12 y	3	skin and itch improvement	
		NCT01986933	18–65 y	2	itch improvement	
		NCT03921411	12–17 y	2	not concluded	
		NCT04921345	7–11 y	2	Recruiting	
REGN3500	IL-33	NCT03736967	18–75 y	2	skin improvement	
		NCT03738423	18–75 y	2	skin improvement	
Fezakinumab	IL-22	NCT01941537	18–75 y	2	skin improvement	
Tezepelumab	TSLP	NCT03809663	18–75 y	2	skin improvement	
MOR106	IL-17	IGUANA	18–65 y	2	NA	
Secukinumab	IL-17	NCT03568136	≥18 y	2	skin improvement	
		NCT02594098	18–85 y	2	NA	
Ustekinumab	Il-12, IL-23	NCT01945086	20–65 y	2	not significant skin improvement
		NCT01806662	18–75 y	2	not significant skin improvement
**oral JAK inhibitor**
Upadacitinib	JAK1	AD Up	12–75 y	3	skin improvement	
		Measure Up 1	12–75 y	3	skin improvement	
		Measure Up 2	12–75 y	3	skin improvement	
		Rising Up	12–75 y	3	NA	
		NCT02925117	18–75 y	2	skin improvement	
Abrocitinib	JAK1	JADE MONO-1	≥12 y	3	skin improvement	Japan for adults and adolecents with moderate-to-severe AD (173)
		JADE EXTEND	≥12 y	3	NA	EU for adults with moderate-to-severe AD (174)
		JADE MONO-2	≥12 y	3	skin improvement	
		JADE Compare	≥18 y	3	skin improvement	
		JADE TEEN	12–17 y	3	NA	
Gusacitinib	JAK, SYK	RADIANT	18–75 y	2	NA	
Baricitinb	JAK1, 2	BREEZE-AD1, AD2, AD3, AD4, AD7	≥18 y	3	skin improvement	EU for adult with moderate-to-severe AD (2020) (175)
		NCT02576938	≥18 y	2	skin improvement	
		NCT03952559	2–17 y	3	Recruiting	
**topical JAK inhibitor**
Ruxolitinib	JAK1, 2	TRuE AD1, AD2	≥12 y	3	skin improvement	FDA for adults with mild to moderate AD (176)
Tofacitinib	JAK1, 2, 3	NCT02001181	18–60 y	2	skin improvement	
Ifidancitinib	JAK1, 3	NCT03585296	≥18 y	2	skin improvement	
Delgocitinib	JAK1, 2, 3/Tyk2	JapicCTI-184064	≥2 y	1	NA	Japan for children with moderate to severe AD (2020) (177)
	JAK1, 2, 3/Tyk2	DELTA 1	≥18 y	3	skin improvement	
	JAK1, 2, 3/Tyk2	DELTA 2	≥18 y	3	NA	
	JAK1, 2, 3/Tyk2	NCT03725722	≥18 y	2	skin improvement	
Brepocitinib	JAK1/Tyk2	2018-003050-24	12–75 y	2	NA	

NA, not available; y, years; m, months; Janus kinases (JAK) inhibitors; CSU, chronic spontaneous urticaria; FDA, Food and Drug Administration; EMA, European Medicines Agency; EU, European Union.

Targeted therapy advantages are related with its high specificity for one or few molecules, and therefore with its low toxicity compared to other systemic therapy for AD. This approach could be translated in IEIs-A with skin involvement in which such pathways are also affected. IEIs-A include a wide variety of diseases with different severity and prognosis and HSCT is the therapy of choice in a large number of these disorders, for which this treatment is potentially curative. In IEIs-A with immunodeficiency requiring HSCT, the use of biologics should not delay timing of transplantation. Indeed, biologics have proved to be effective in modulating an altered pathway and relieving symptoms but do not represnet a definitive therapy in immunodeficiencies. Rare diseases such as IEIs are inherently difficult to study in well-controlled clinical trials and therefore need a multidisciplinary management involving clinical immunologists and dermatologists to perform correct diagnosis and appropriate therapy in order to improve patient outcomes.

## 6. Conclusions

IEIs management is challenging but still affordable when a prompt diagnosis and an appropriate treatment are established. A diagnostic delay of IEIs is historically reported due to the variability of clinical phenotype and their rarity. However, the increasing availability of NGS technology together with recent research advances in IEIs and IEIs-A have improved the early diagnosis and optimized the treatment of these conditions. The speed, accuracy, and sensitivity of molecular analysis is crucial in the era of precision medicine based on a person's disease-driving molecular alterations. Biologics have the great advantage to act on a targeted component of immune system and they are becoming increasingly effective and safe for the therapeutic approach of many skin diseases.

There is an essential lack of knowledge about the efficacy of biologics in IEIs-A and only limited case reports describing their use in clinical practice are available. Long-term follow-up studies need to assess the safety and persistence of efficacy of each biologic.

More and larger international multicenter studies in this special population are necessary to evaluate the clinical profile of new drugs and to identify biological markers which will help to select patients who may benefit from tailored interventions.
